# Anti-Allergic and Anti-Inflammatory Signaling Mechanisms of Natural Compounds/Extracts in In Vitro System of RBL-2H3 Cell: A Systematic Review

**DOI:** 10.3390/cells13161389

**Published:** 2024-08-21

**Authors:** Tekan S. Rana, Rishipal R. Bansode, Leonard L. Williams

**Affiliations:** Center for Excellence in Post-Harvest Technologies, North Carolina Agricultural and Technical State University, North Carolina Research Campus, Kannapolis, NC 28081, USA; tsrana@aggies.ncat.edu (T.S.R.); rbansode@ncat.edu (R.R.B.)

**Keywords:** RBL-2H3, MAPK, FCεRI, allergy, food allergy, signaling pathway, JAK/STAT, NF-κB

## Abstract

Various extracts are tested for anti-allergic or anti-inflammatory properties on in vitro models. RBL-2H3 cells are widely used in allergic or immunological studies. FCεRI and its downstream signaling cascades, such as MAPK, NF-κB, and JAK/STAT signaling pathways, are important allergic or inflammatory signaling mechanisms in mast and basophil cells. This systematic review aims to study common signaling pathways of the anti-allergic or anti-inflammatory compounds on RBL-2H3 cells. We selected the relevant research articles published after 2015 from the PubMed, Scopus, Science Direct and Web of Science databases. The risk of bias of the studies was assessed based on the modified CONSORT checklist for in vitro studies. The cell lines, treatments, assay, primary findings, and signaling pathways on RBL-2H3 cells were extracted to synthesize the results. Thirty-eight articles were included, and FCεRI and its downstream pathways, such as Lyn, Sky, PLCγ, and MAPK, were commonly studied. Moreover, the JAK/STAT pathway was a potential signaling mechanism in RBL-2H3 cells. However, the findings based on RBL-2H3 cells needed to be tested along with human mast cells to confirm its relevance to human health. In conclusion, a single plant extract may act as an anti-inflammatory reagent in RBL-2H3 cells via multiple signaling pathways besides the MAPK signaling pathway.

## 1. Introduction

Allergy is an immunological disorder due to non-toxic environmental factors such as pollen, food, dust, drugs, insect venom, latex, hormones, fungal spores, and vaccines [1,2,3,4,5,6,7]. Allergies are increasing across developed and developing countries [1,2,6,8,9,10], affecting about 20% of the global population [11,12]. The four types of allergies or hypersensitivities are anaphylactic, cytotoxic, immune complex, and delayed, which are called type I, II, III, or IV, respectively [8,13,14]. Type I allergies (e.g., food allergies, asthma, and allergic rhinitis) are the most common [3,4,8,11,13].

How do type I allergies begin? There are two stages: initially, when an allergen is exposed for the first time, which is referred to as the sensitization or induction phase [15], antigen-presenting cells (APCs) or macrophages recognize, engulf, and present it to naïve T cells, which will differentiate into T helper 2 (Th2) cells. These Th2 cells produce pro-inflammatory cytokines such as interleukin-4 (IL-4), IL-5, or IL-13, which convert B cells into IgE-producing cells (i.e., plasma cells). The produced IgE binds with the α-subunit of high-affinity IgE receptors (FCεRIs) of mast and basophil cells [3,16], which are primary effector cells in type I allergy [14]. When the same allergen is present again, it is called the effector phase [15]. The allergen will crosslink two adjacent FCεRI -bound IgE, triggering various downstream signaling cascades such as tyrosine kinase, protein kinase C (PKC), mitogen-activated protein kinase (MAPK) [3,12,17], Janus kinase-signal transducer and activator of transcription (JAK/STAT), and nuclear factor κB (NF-κB) [9], as well as calcium influx [12] and cytoskeleton remodeling, recruiting secondary cells such as neutrophils [15], causing degranulation (i.e., histamine and β-hexosaminidase release), the production of reactive oxygen species (ROS) [18], and various pro-inflammatory cytokines and chemokines production [2,3,4,6,11,13,17,19,20,21,22,23].

**Figure 1:**
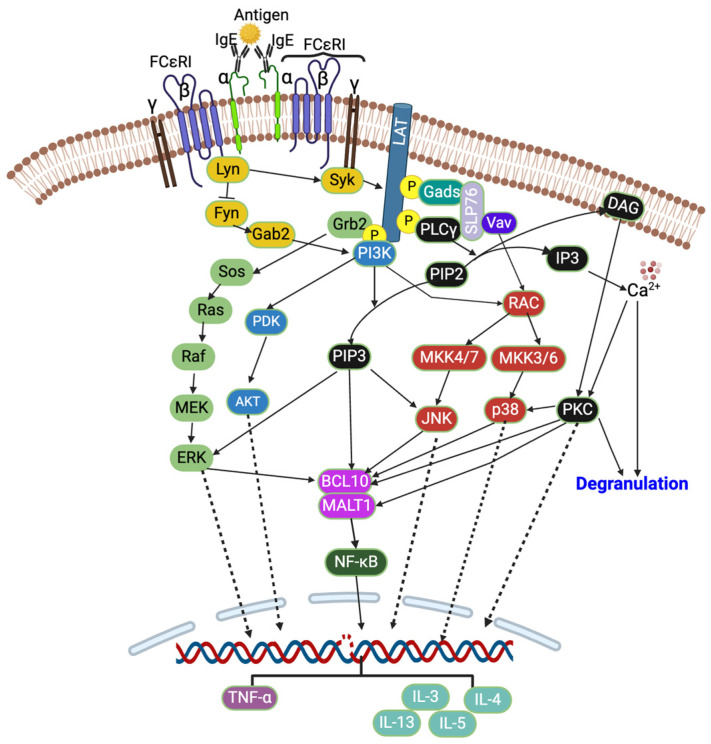
FCεRI signaling pathway. Created with BioRender.com.

The crosslinking of IgE by an allergen activates the heterotetrameric (one α, one β, and two γ subunits) FCεRI receptors, which further activate two Src family kinases, Lyn and Fyn, which are protein tyrosine kinases (PTKs) (Figure 1). These Lyn and Fyn activate FCεRIβ-immunoreceptor tyrosine-based activation motifs (ITAMs) and recruit spleen tyrosine kinase (Syk) to FCεRIβ-ITAMs [14,24]. The Syk further activates other signaling cascades such as PKC, protein kinase B (Akt), phosphoinositide 3-kinase (PI3K), rat sarcoma (Ras), guanosine triphosphatase (GTPase), and phospholipase Cγ (PLCγ) [13,16,17,20,25,26]. The activated Syk also activates linkers for the activation of T cells (LAT) and src homology 2 (SH2) domain-containing leukocyte-specific phosphoproteins of 76 kd (SLP-76). This is followed by the event where cytosolic adaptor molecules such as growth factor receptor bound protein 2 (Grb2), glutamic acid decarboxylase 2 (Gad2), PLCγ1, and guanine exchange factors (VAV and SOS) bind to LAT, and this further activates PI3K and MAPK signaling pathways [14,24]. The PLCγ converts phosphatidylinositol 4,5-bisphosphate (PIP2) into diacylglycerol (DAG) and inositol triphosphate (IP3), which further reduces the intracellular Ca^2+^ [27,28]. The Ca^2+^ and DAG activate PKC [14,28], which then activates p38 MAPK, B cell lymphoma/leukemia 10 (BCL10), and mucosa-associated lymphoid tissue lymphoma translocation protein 1 (MALT1) [28]. The activated PI3K converts PIP2 into phosphatidylinositol 3,4,5-triphosphate (PIP3), and PIP3 further activates JNK (via RAC and MAPK4) and ERK1/2 (Figure 1). The ERK1/2 activates phospholipase A2 (PLA2), increasing leukotriene and prostaglandins [28].

**Figure 2:**
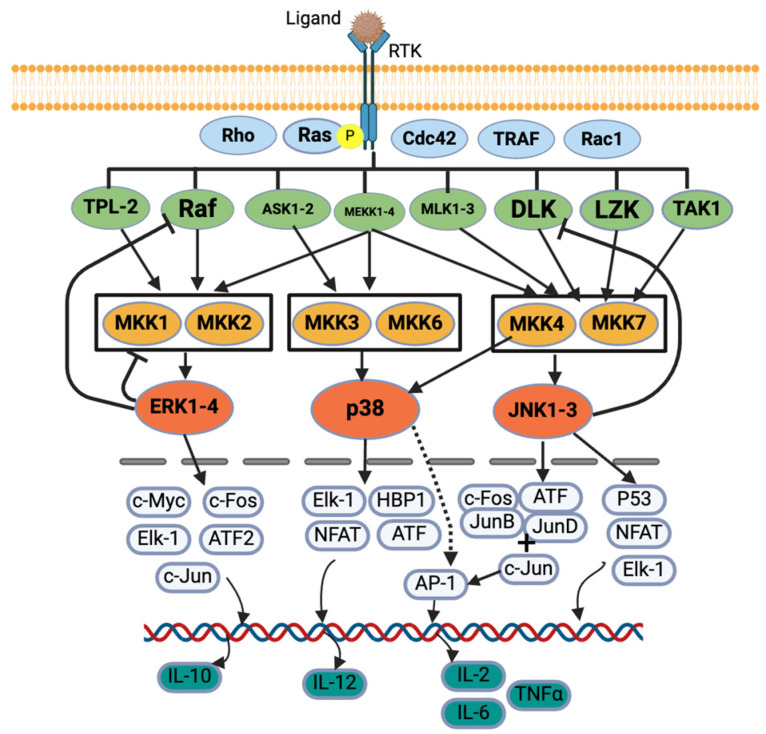
MAPK signaling pathway. Created with BioRender.com.

MAPK and JAK/STAT are the most important signaling pathways for allergies [9,25]. Five protein kinases’ sequential activation regulates the MAPK signaling cascade: MAPK kinase kinase kinase (MAP4K), MAPK kinase kinase (MAP3K), MAPK kinase (MAPKK), MAPK, and MAPK-activated protein kinases (MAPKAPK). In general, MAP3K, MAP2K, and MAPK are commonly explained in studies [29,30]. MAPK has four categories based on their structure, activation motif, and function: extracellular signal-regulated kinase 1/2 (ERK1/2), p38 MAPK, c-Jun N terminal kinase 1/2 (JNK1/2) [9,11,12,30], and ERK5 (not commonly explained) [29]. While pro-inflammatory stimuli activate all categories of MAPK, growth factors and hormones activate ERK1/2, and cellular and environmental stresses activate p38 MAPK and JNK 1/2 [30] (Figure 2).

In the canonical activation of the ERK1/2 MAPK cascade, a ligand first binds to a receptor tyrosine kinase (RTK), activating a G-protein, Ras. The Ras recruits and activates serine/threonine protein kinase, Raf (a MAP3K), which activates MAPK/ERK kinases (MEK1/2) (a MAP2K also known as MKK1/2), and these MEKs further activate ERK1/2 (a MAPK) [29,30,31,32] (Figure 2). ERK1/2 also provides negative feedback to the proteins, e.g., SOS protein, Raf-1 and MEKs, upstream of the signaling cascades [29]. Once translocated into the nucleus, the ERK regulates various transcription factors such as c-Fos, c-Jun, c-Myc, Elk-1, and ATF2 [29]. The ERK regulates IL-10 production, which helps Th cells to Th2 cells producing IL-4, -5, -9, and -13 [30] (Figure 2). Syk also activates ERK1/2, which further activates the arachidonic signaling pathways and production of TNF-α, IL-2, IL-5, and IL-13 in mast cells.

For p38 MAPK activation, tumor necrosis factor receptor-associated factor 2/3/6 (TRAF) or Rho protein activates MEKK1, SAK1, or TAK1 (a MAPK3K) in response to stress or cytokines. They activate MKK3 or MKK6 (a MAP2K), which finally activates p38 MAPK [30,32,33,34]. The p38 further regulates the transcription factors such as ATF, NFAT, Elk-1, and HBP1, which further regulates the cytokine production [34] (Figure 2). The p38 MAPK regulates IL-12 production, which promotes Th cell differentiation into Th2 cells, which produce IL-2, IFN-γ, and TNF-α/β [30]. 

Diverse biotic and abiotic stresses, including cytokines (e.g., TNF and IL-1), activate the JNK pathway via various receptors such as TNFR, GPCR, TGFBR, and TLR [35]. Various signals activate Rac1/Cdc42, which activates their downstream proteins such as MLK, ASK, DLK, MEKK, and TAK. They further activate MKK4 or MKK7, which ultimately activates JNK [32,33]. JNK also provides negative feedback to an upstream protein, DLK [35]. The JNK further acts on various transcription factors such as c-Fos, ATF, Jun B, Jun D, and c-Jun, which activates AP-1 that regulates cytokine production [32,33,35]. The JNK also regulates P53, NFAT, and Elk-1 [32] (Figure 2). The p38 MAPK activates IL-4, and the JNK activates IL-2, IL-6, and TNF-α [9,25].

**Figure 3:**
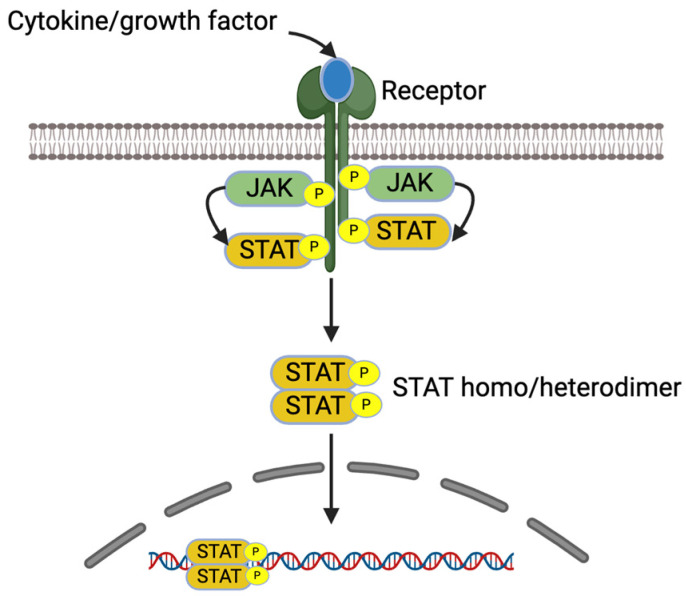
JAK/STAT signaling pathway. Created with BioRender.com.

IL-4 and IL-13 increase pro-inflammatory gene expression in allergic diseases via JAK/STAT signaling pathways [7]. These cytokines bind receptors, which lead to receptor dimerization and the recruitment of Janus kinase (JAK). The activated JAKs activate the receptors and recruit STATs to the receptors. The activated STATs dissociate from the receptors as homo or heterodimers and are translocated into the nucleus, bind into DNA, and regulate the gene expression [7,36] (Figure 3).

MAPK can activate NF-kB [28]. Inactive NF-kB in cytoplasm is found in trimeric form with an NF-kB inhibitor (IkB). Signals from MEKK1 activate the IkB kinase complex (IKK) [37], which further activates IkB (at Ser 32 and Ser 36 residues), releasing NF-kB into the nucleus where it binds to the kB binding site of promotor regions and activates gene expression and mediators—for example, COX-2, TNF-α, IL-1β, -6, and -8 [38,39,40]. The activated NF-kB presents in the heterodimer form of p65 and p50 subunits where the activated IkB is ubiquitinated by the 26S proteasome [39].

Mast cells are vital for allergic reaction, as their activation leads to the release of mast cell mediators such as histamine, leukotriene C4 (LTC4) and prostaglandin D2 (PGD2), causing early reaction. Other mast cell mediators such as IL-3, IL-5, IL-8, and tumor-necrosis factor (TNF) recruit eosinophils, neutrophils, and Th2 cells, and they also interact with other tissue cells to start late-phage allergic reaction [41]. Since mast cells regulate allergic reactions in a multi-faceted way, anti-allergic drugs to control the allergic reaction due to mast cells could be used at various levels such as (a) targeting mast cell mediators and their receptors, (b) mast cell activators, their receptors, and signal transduction, (c) mast cell inhibitory receptors, and (d) reducing mast cell numbers [42]. In other words, mast cells can be targeted with anti-allergic drugs at three levels: the cell membrane (e.g., Omalizumab, which targets free IgE and reduces the IgE attachment to FCεRI), intracellular (e.g., Syk kinase inhibitors, which block IgE-FCεRI-mediated downstream phosphorylation) or extracellular (e.g., H1–4 receptor antagonists which prevent binding and the effect of histamine on target cells) [43,44]. However, since mast-cell-derived mediators are also produced by other cells, targeting those mediators would not provide the answer to the absolute necessity of mast cells for the reaction. On the other hand, mast cell activating receptors are unique to those cells, and blocking them, for example by using anti-IgE antibodies omalizumab and ligelizumab, could provide the definite role of mast cells for the reaction [42]. These monoclonal antibodies block the attachment of IgE to FCεRI on mast cells and indirectly reduce the FCεRI expression in the cell, resulting in downstream signaling reduction [43].

The rat basophilic leukemia cell (RBL-2H3) line is commonly used for various allergic and immunological studies, specially to study the IgE-FCεRI-mediated signaling mechanism [8,10,12,17,23,45], as it has similar characteristics (such as the presence of histamine, β-hexosaminidase, and the expression of FCεRI) [46] and response behavior as those of mast cells and basophil cells [2,10,21,22,25,47]. However, detailed information on RBL-2H3’s potential to test multiple signaling pathways/mechanisms of various anti-allergic or anti-inflammatory compounds or extracts on RBL-2H3 cell lines is scarce. Thus, this systematic review aims to study the common signaling pathways of anti-allergic and anti-inflammatory compounds in RBL-2H3 cells.

## 2. Materials and Methods

**Figure 4:**
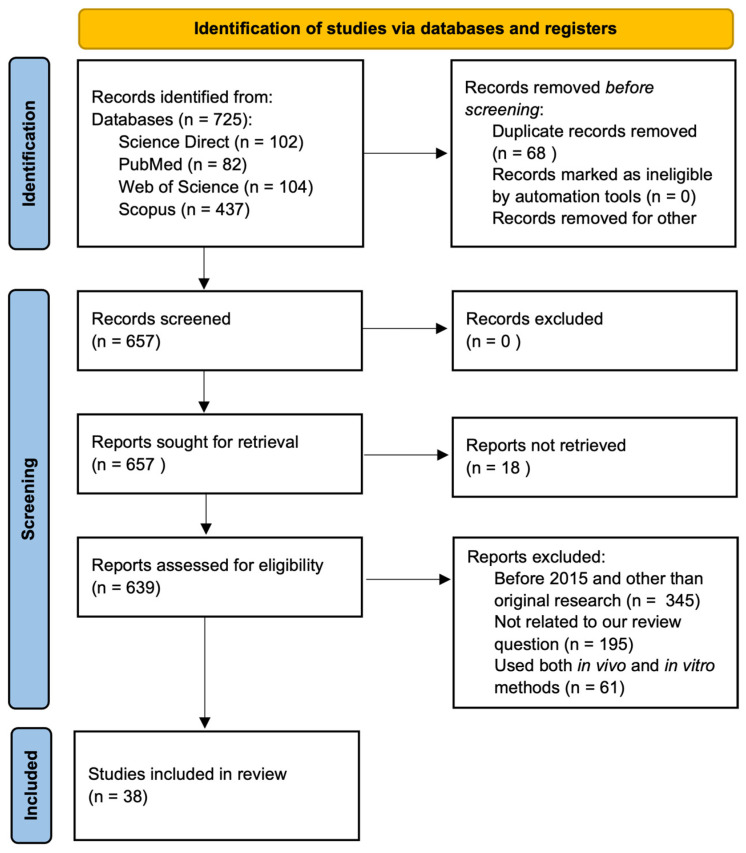
PRISMA flow diagram showing literature search and selection process.

This systematic review was conducted according to Preferred Reporting Items for Systematic Reviews and Meta-analysis (PRISMA) guidelines [48], and the PRISMA flow chart shows the detail of the articles’ selection process and search results (Figure 4). Out of the 657 total articles, 18 were not fully retrieved, 345 were published before 2015 and were not original research articles, 195 were not closely related to the topics, and 61 included both in vitro and in vivo systems, leading to the final 38 articles for the review.

We searched literature utilizing four databases: PubMed, Scopus, Science Direct, and Web of Science until 5 March 2024. We used the following keywords, in the same order, in Scopus and Science Direct: (“RBL-2H3”) AND (MAPK OR “JAK/STAT” OR “NFkB” OR “FCεRI” OR “signaling pathway”) AND (“allergy” OR “peanut allergy”) AND (polyphenol). In Web of Science, all keywords mentioned above were identical except that “Polyphenol” was omitted. For PubMed, we used following keywords: ((“RBL-2H3”) AND (MAPK OR “JAK/STAT” OR “NFkB” OR “FCεRI” OR “signaling pathway”) AND (“allergy” OR “peanut allergy”)) and the following Medical Subject Headings (MeSH) separately: ((((rbl-2h3) OR (“Mast Cells” [Majr])) AND (“Signal Transduction” [Majr])) AND (“Hypersensitivity” [Majr])). Two authors (T.S.R. and R.B.) screened the articles, and any disagreements were solved through discussion and consultation with a third author (L.L.W.).

The PICO framework was used to determine the evidence in the articles as follows: population: RBL-2H3 cells only (or along with other cell lines); intervention: natural compounds/extracts; comparison: extract/compound-treated cells versus untreated cells; outcome: signaling pathways, cell degranulation, and inflammatory cytokines.

The articles were deduplicated by uploading them in Excel sheets followed by the title and abstract screening for inclusion/exclusion criteria and were further evaluated on full text for their eligibility. Two reviewers (T.S.R. and R.R.B.) independently screened the title and abstract for the inclusion. The original research articles using an in vitro system, published in the English language in peer-reviewed journals from 2015 to 2024 (5 March), were included for the review. Review articles, proceedings, or articles published in languages other than English, articles published before 2015, articles not closely related to the topic (i.e., those that did not include RBL-2H3 cells, pro-inflammatory cytokines, or signaling pathways/molecules), and articles that included both in vitro and in vivo systems were not selected in this review. We excluded studies that used both in vitro and in vivo systems to focus our review on an in vitro system using RBL-2H3 and other cells in order to avoid variability and complexity in our study. Two authors (T.S.R. and R.R.B.) agreed upon the selection criteria and on selected articles.

The following items were manually and independently extracted and agreed by two authors (T.S.R. and R.R.B.) from the selected articles: publication year, objectives, cell lines used, dose and duration of the treatment/control, sequence of sensitization, treatment and stimulation, assay/techniques used, primary findings, and treatment effect on signaling pathways/molecules on RBL-2H3 cells. When information on signaling pathways were not provided in the included articles, cell degranulation signature molecules such as histamine and β-hexosaminidase and cytokines expression data were obtained. Moreover, the compounds’ extraction methods were not included from the selected articles.

The risk of bias of selected articles was independently assessed and agreed by two authors (T.S.R. and R.R.B.) using a modified CONSORT checklist for in vitro study [49,50]. The comprehensiveness and unbiased reporting on the following items of the articles were assessed: abstract, background and rationale, objectives, hypothesis, intervention, outcome, statistical method, outcome estimation, limitations, and funding. As the articles were diverse in terms of treatment used, objectives, and design, we used a narrative synthesis approach to synthesize the articles. The information were tabulated according to cell line used, i.e., either only RBL-2H3 or RBL-2H3 along with other cell lines. The synthesis steps were conducted by two authors (T.S.R. and R.R.B.) and agreed among all three authors (T.S.R., R.R.B., and L.L.W.).

## 3. Results

### 3.1. Study Characteristics, Risk of Bias, and Reporting Quality

**Figure 5:**
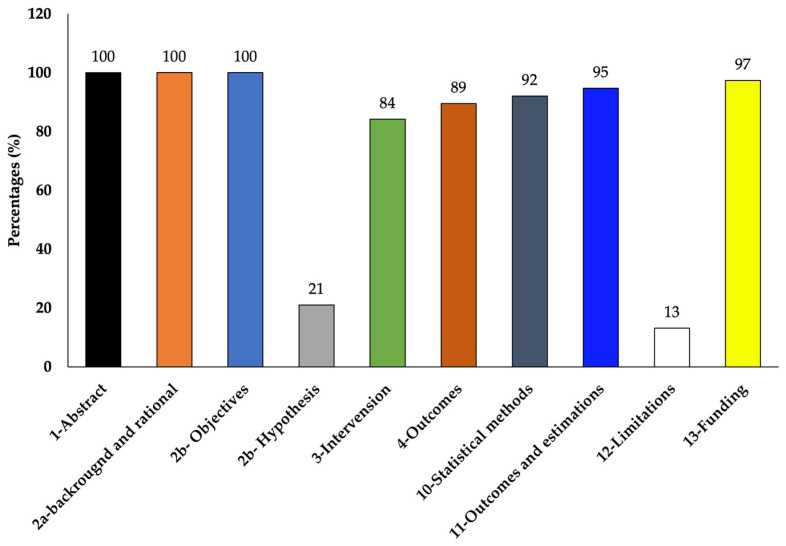
Percentages of the reviewed articles that met the modified CONSORT checklist.

The selected studies varied in their objectives and overall experimentation. Out of the total of 38 chosen studies, 26 studies used only RBL-2H3 cells and 12 studies used RBL-2H3 along with other cells such as RAW264.7 (5 studies), HaCaT (2 studies), HiMC (1 study), BMMC (1 study), Caco2 (1 study), MoLT-4 (1 study), HMC-1 (1 study), KU812 (1 study), human neutrophils (2 studies), human basophils (1 study), and bacteria (Klebestella pneumonia) (2 studies) (Table 1 and Table 2). According to the CONSORT checklist used [49,50], although most of the studies did not mention the hypothesis and limitation of their studies clearly, the majority of them clearly explained the experimental details so that the experiment could be replicated and also reported complete results showing quality results with low risk of bias (Table 3 and Figure 5).

### 3.2. Signaling Pathways

Data extracted from the included studies are summarized in Table 1 and Table 2. Studies had diverse compounds tested for their anti-allergic or anti-inflammatory properties and possible signaling mechanisms. For example, studies including only RBL-2H3 had extracts from plants, shrimps, fungi, sponges, and chemicals (sodium sulfite) (Table 1). However, the majority of the studies were related to plant extracts. Similarly, studies those used multiple types of cells also had all plant extracts except one study with enzymatically synthesized glycogen (Table 2). Some studies did not explore the signaling mechanisms of the tested compounds in depth. In contrast, most of the studies had shown that the anti-allergic or anti-inflammatory activities of tested compounds on RBL-2H3 were via the regulation of MAPK, FCεRI, NF-kB signaling pathways, or their upstream or downstream regulators such as Syk, Lyn, Akt, PLCγ, PI3K, PKC, and LAT (Table 1 and Table 2). In addition to FCεRI and its downstream signaling, some studies have also shown that RBL-2H3 cells demonstrate JAK/STAT and NLRP3 signaling pathways when they were tested for anti-allergic mechanisms of fungus extract and sodium sulfite, respectively (Table 1). Moreover, the studies had shown that a single compound can have multiple signaling pathways (Table 1 and Table 2).

## 4. Discussion

This review has the following limitations. (i) We used peer-reviewed articles published in the English language, which may have excluded important results published in other languages and in preliminary result reports. (ii) We only included in vitro studies and articles published after 2015. (iii) We did not manually search and include the literature from the included articles’ reference lists. (iv) There are no standard methods on quality evaluation of the in vitro studies, so some modified methods recommended for the clinical setting were used. These shortcomings prevent the comparison of in vivo and in vitro results and the relevance of in vitro studies’ findings for further clinical trials. Moreover, exclusion criteria may lead to missing valuable sources regarding the use of RBl-2H3 for novel signaling mechanisms for allergic or immunological research. Furthermore, the lack of a standard protocol for in vitro study quality may also result in variations in the articles’ quality. However, this review provides a trend on the use of RBL-2H3 cells and their potential signaling mechanisms for allergic and inflammatory studies over a decade.

The studies had diverse compounds tested, such as a variety of extracts from plants (on the majority of included articles), fungi [9,21], shrimp [56], sponges [20], agro-industrial waste [6,53,54], and chemical compounds [55]. Thus, there is also a need for multiple studies using the same or similar compounds, which may be met by broadening the literature inclusion criteria. Moreover, the included studies primarily studied MAPK signaling pathways, and other critical pathways such as JAK/STAT pathways were barely reported. This might be related to the fact that RBL-2H3 cells are not exactly the same as mast cells or basophil cells. For example, studies have reported that RBL-2H3 cells lack some properties of mast cells, such as the absence of essential elements such as CD14 and MyD88. However, they have Toll-like receptor 4 (TLR4) on their surface [64], which might have limited the cells from being used in TLR-related signaling pathways such as JAK/STAT pathways. However, some studies suggested that the presence of CD14 does not guarantee that the TLR-acting stimulus, such as LPS, directly activates the cells [65]. However, some of the included studies had reported STAT6 signaling pathways [9] and NLRP3 and caspase pathways [55] in RBL-2H3 cells, indicating their potential use for diverse signaling mechanisms. Moreover, studies related to new applications of tested compounds/extracts, such as using them as a nutraceutical or in industry-standard applications, still need to be included.

From this study, it is also evident that the dose and duration of the tested compound can impact the level of expression or presence and absence of the tested signaling pathways. The literature also reported the variations on the RBL-2H3 phenotype based on various factors such as cultural condition [47,64], age, and biological sex from which the cell line was isolated [46]. Thus, it is important to provide details of the used cell’s properties (e.g., cell passage, catalog number) in the studies for their reproducibility.

Despite the mast cells and basophil cells being similar to RBL-2H3 in many properties, we excluded them in this review because RBL-2H3 cells still differ from those cells in various characteristics such as the amount of histamine in the cells, the absence of CD14, TLR-2, the presence of c-kit, which is a receptor for stem cell factor (SCF) [65], the absence of CD123, which is a signature of primary basophil, and not expressing rat tryptase genes and release tryptase, which are found in primary mast cells [46].

It is also essential to critically consider the relevance or implication of RBL-2H3 studies’ results to human mast cells. There are various limitations on the use of human mast cells or basophil cells for studies. For example, the isolation and purification of human mast cells and basophil cells is expensive, tedious, impractical, and results in issues in the isolated cells’ purity and viability [41,66]. Moreover, various human mast cell lines have been developed but suffered from vital issues over time. For instance, HMC-1 and HMC-α failed to degranulate following immunological activation; LAD-2 has a high cost and slow growth rate, so it is not the first choice for routine experimental studies; ROSA ^KIT D816V^ does not have histamine and β-hexosaminidase expression; and ROS ^KIT WT^ needs stem cell factor (SCF) to survive, which is very expensive and not affordable for routine studies [41,66]. Due to these limitations for the use of actual human mast cells, the use of RBL-2H3 cells becomes prominent, as these cells are easy to culture, have homogeneity with a rapid growth rate, have functional FCεRI, and have similar degranulation dynamics to human mast cell and basophil cells [41,66]. However, despite some similarities between RBL-2H3 cells and the mast cell (e.g., both have c-kit receptors for SCF), there are differences, too. For example, unlike in mast cells, the TLR4 or its co-receptor CD14 and TLR2 are not expressed in RBL-2H3 [47,65]. Moreover, since RBL-2H3 cells are immortalized cells, their physiology might not be the same as that of primary mast cell physiology, and RBL-2H3 cells also show intra-laboratory reproducibility issues. Thus, the results obtained with these cells should be validated along with the human mast cell lines [41,46,47,66].

In the included studies, other types of cells (not RBL-2H3) were used to study the antioxidant properties, pro-inflammatory cytokines, or gene-level expression of some allergic markers. Moreover, the crude extract versus purified compound may have affected the expression of signaling pathways on RBL-2H3 cells. The variation in the dose and time of the tested compounds and sensitizing/stimulating agents such as anti-DNP-IgE (33.3 ng/mL for 2 h—1 μg/mL for overnight), ionomycin (1 μM for 30 min–250 μM for 30 min), DNP-BSA (50 ng/mL for 1 h—10 μg/mL for 30 min), calcium ionophore A23187 (0.15 ug/mL for 30 min—1 μM for 2.5 h), and DNP-HSA (5 ng/mL for 1 h—100 ng/mL for 18 sec) (Table 1 and Table 2) may also contribute to variation in the signaling mechanisms of the tested compounds on RBL-2H3 cells.

## 5. Conclusions

Most of the compounds tested on RBL-2H3 cells had shown that FCεRI and MAPK signaling pathways are dominant for anti-allergic and anti-inflammatory mechanisms. Moreover, due to variations in the types of the tested compounds, it is also revealed that a single compound can affect various stages of multiple signaling pathways. The RBL-2H3 cells should have cytokine receptors (required for JAK/STAT signaling) considering their similarities with basophil and mast cells which have cytokine receptors in their cell membranes [41]. Moreover, a fungus extract called viridicatol treatment on RBL-2H3 had reduced the STAT6 expression [9]. Thus, RBL-2H3 may also be able to be tested for JAK/STAT signaling pathway for plant extracts.

## Figures and Tables

**Table 1:** Anti-allergic and anti-inflammatory properties and signaling pathways of various compounds using only RBL-2H3 cells.

Ref.	Objectives	Methods: Treatment; Control	Methods: Dose (Duration)	Method: Sensitization, Treatment, Stimulation Sequence	Methods: Assays	Main Findings	Findings: Signaling Pathways in RBL-2H3 Cells
Bansode et al., 2018 [51]	Anti-allergic activity of PSP-enriched PN protein aggregate	PSP-PN aggregates; peanut flour w/o ionomycin (control)	Anti-DNP-IgE @ 1 µg/mL (overnight); PN: PSP aggregate (0–40% PSP: PN flour ratio, w/w), the protein exposure level @ 100 μg/mL; DNP-BSA @ 1 μg/mL + ionomycin @ 1 μM (3 h)	IgE > Tmt > DNP-BSA+ Ionomycin	WB, ELISA, histamine assay, β-hex assay	30% PSP-PN ↓ β-hex (54.2%) and histamine (49.2%). 40% PSP-PN ↓ IgE binding by 19%. PSP-PN aggregates ↓ p44/42 MAPK, but ↑ p38 MAPK and SAPK/JNK	↓ MAPK p44/42
Barbosa et al., 2018 [2]	Anti-allergic property of phlorotannin-targeted extract of seaweed	Phlorotannin-targeted extracts; Quercetin (+ve)	Extract @ 125–500 μg/mL (30 min); A23187 @ 150 ng/mL (30 min); OR Anti-DNP IgE @ 50 ng/mL (16 h); DNP-BSA @ 50 ng/mL+ dry extract @ 125–500 μg/mL (1 h)	Tmt > A23187; IgE > DNP-BSA + Tmt	MTT reduction assay, CVS assay, HAase inhibition assay, β-hex assay	Extracts ↓ β-hex (79% to 31% ↓ at 500 μg/mL extract) and histamine (67 to 55% ↓ at 500 μg/mL extract) released in a dose-dependent manner	NA
Chang et al., 2015 [3]	Beneficial effect of the Monascin and ankaflvin	Monascin, ankaflavin, rosiglitazone, and GW9662 (PPAR*γ* antagonist); Ionomycin/PMA (+ve), 15% FBS (MEM) (-ve)	Monascin, ankaflavin, rosiglitazone (each @ 40 μM) (24 h); PMA @ 50 nM PMA + ionomycin @ 500 nM (3 h). For β-hex, treated cells > ionomycin @ 10 μM (30 min)	Tmt > PMA + Ionomycin	CVS assay, WB, ELISA, β-hex assay	Forty micromolar monascin and ankaflavin ↓ PMA/ionomycin-induced mast cell degranulation and TNF-α secretion through ↓ PKC and MAPK family (ERK, JNK, and p38)	↓ MAPK (ERK, JNK, and p38) and PKC
Dippenaar et al., 2022 [52]	Develop a protocol for Honeybush extract to yield fractions with higher anti-allergy potential	Hot water extract, four fractions on XAD 1180N; MEM (−ve), Wortmannin (W) (+ve)	Anti-DNP-IgE @ 500 ng/mL (24 h); extract @ 62.5–250 μg/mL or W @ 100 nM (30 min); DNP-hSA @ 5 ng/mL (1 h)	IgE > Tmt > DNP-hSA	β-hex assay, SARS assay, DPPH scavenging assay, ORAC assay, XOI assay	Fraction 1 ↓ the β-hex activity compared to extract	NA
Gaihre et al., 2022 [53]	Perilla pomace’s (i.e., Egoma) function in allergy	Egoma extracts (four cultivars); Tyrode buffer (control)	Anti-DNP-IgE @ 33.3 ng/mL (2 h); Tyrode’s buffer +/− extract (10 min); 30 μL (for β-hex) or 150 μL of DNP-HSA @ 200 μg/mL (1 h)	IgE > Tmt > DNP-HSA	CCK-8 assay, WB, HPLC, β-hex assay	Compared to the control, extracts from two Japanese cultivars ↓ β-hex, Syk, PLC*γ*2, and Akt proteins	↓ Syk, PLC*γ*2, and Akt
Hamauzu et al., 2021 [54]	Anti-allergic effect of fruit extracts and agro-industrial by-products	Hot water and ethanol extracts of mature fruits, immature fruits, liquor residue, lima bean pod, and pomace	Extracts @ 100 μg/mL (24 h); anti-DNP IgE @ 1 μg/mL (2 h); DNP-hSA @ 10 ng/mL (30 min)	Tmt > IgE > DNP-hSA	β-hex assay, TPC assay, proanthocyanidin assay, HPLC, pectin content assay, bile acid-binding assay	Grape bunch stem ethanol extract had the highest ↓ (63.6%) on β-hex. No correlation between TPC and anti-degranulation property, +ve correlation between proanthocyanidin and degranulation. Higher proanthocyanidin content (100 mg GAE/g dry extract) adversely affected degranulation	NA
Jiao et al., 2017 [20]	The anti-allergic potential of dysivillosins A–D (form Marine sponge)	Dysivillosins A–D; ketotifen (+ve)	DNP-IgE @ 1 μg/mL (overnight); dysivillosins A–D @ 6 and 12 μM (30 min); DNP-BSA @ 1 μg/mL (1.5 h)	IgE > Tmt > DNP-BSA	MTT assay, WB, ELISA, β-hex assay	All compounds dose-dependently ↓ β-hex, IL-4, and LTB4. Dysivillonsin A was themost potent and reduced PLC*γ*1 and Syk in a dose-dependent manner	↓ PLC*γ*1, Syk
Kawai et al., 2018 [21]	Anti-allergic effect of *Grifola frondosa* mushroom extract (GFE)	GFE; DNP-hSA (-ve), Tranilast (+ve)	Anti-DNP IgE @ 10 μL/mL (24 h); DMSO (0.5%) or ethanol (1%) dissolved extracts @ 10–50 μM (20 min); DNP-hSA @ 20 ng/mL (30 min) and β-hex and histamines measured. For mRNA detection, the anti-DNP IgE @ 10 μL/mL (24 h); ergosterol @ 50 ng/mL (20 min or for 0–10 min for phospho-tyrosine); DNP-hSA @ 20 ng/mL (2 h) or @ 50 ng/mL (0–10 min) for phospho-tyrosine	IgE > Tmt > DNP-hSA	β-hex assay, histamine release assay, RT-qPCR, WB, immunofluorescent microscopy	The active compounds ↓ β-hex, histamine. Ergosterol ↓ FcεRI aggregation, IL-4, TNF-α mRNA	↓ FcεRI aggregation
Kobayashi et al., 2015 [4]	Cell degranulation property of PMFs from *Kaempferia parviflora*	PMFs; Wortmannin (control for IgE induced β-hex), A23187 and DTBHQ + PMA (control for A23187 induced β-hex)	Anti-DNP IgE @ 50 ng/mL (2 h); whole extracts @ 0–1000 μg/mL or extract components @ 0–100 μM or Wortmannin @ 25 μM (10 min); DNP-hSA @ 50 ng/mL (30 min) and β-hex measured. For A23187 induced degranulation, A23187 @ 10 μL of 250 μg/mL or 10 μL of 500 μM of DTBHQ+ 10 μL PMA (30 min). For PLC*γ*1, PLC*γ*2, Syk, FcεRI protein measurement, the sensitized cells were incubated with KP02 and KP10 @ 100 μM (1 h); DNP-hSA @ 50 ng/mL (1 min)	IgE > Tmt > DNP-hSA; A23187 or DTBHQ + PMA > Tmt> DNP-hSA	β-hex assay, WB	All the flavones ↓ β-hex. The KP02 and KP10 ↓ PLC*γ*1 and Syk. No effect on cytoplasmic FcεRI but ↓ membrane FcεRI	↓ PLC*γ*1, Syk and membrane FcεRI
Lee et al., 2017 [8]	Mechanism of anti-allergic effect of the *Zizania latifolia* extract	Extracts or its fractions; IgE+ DNP-BSA (control)	Anti-DNP IgE @ 450 ng/mL (overnight); 20 μL of extract @ 10–100 μg/mL or the extract fractions @ 0–50 μg/mL (10 min) (or 30 min for COX-2); DNP-BSA @ 20 μL of 10 μg/mL (10 min) (or 4 h for COX-2) and β-hex was measured. For TNF-α, chloroform fraction @ 0–50 μg/mL; A23187 @ 1 μM+ 50 ng/mL PMA (4 h). For protein analysis, anti-DNP IgE @ 450 ng/mL (overnight); chloroform fraction 0–50 μg/mL; DNP-BSA @ 10 μg/mL (15 min)	IgE > Tmt > DNP-BSA; IgE > Tmt > A23187 + PMA	β-hex assay, WB, MTT assay, ELISA	Chloroform faction ↓ β-hex, TNF-*α*, COX-2, and MAPKs	↓ MAPK family (p38, ERK, JNK)
Lee et al., 2020 [25]	Mechanism of tricin and enzyme-treated wild rice *Zizania latifolia* extract (ETZL) on cell degranulation	Tricin or ETZL; DNP-IgE and/or DNP-hSA (control)	Anti-DNP IgE @ 0.05 μg/mL (24 h); tricin @ 10–500 ng/mL or ETZL @ 10–500 μg/mL (1 h); DNP-hSA @ 0.1 μg/mL (4 h) (or 10 min for ERK, AKT, p38, JNK proteins)	IgE > Tmt > DNP-hSA	β-hex assay, WB, MTT assay, ELISA	Tricin and ETZL ↓ β-hex, TNF-*α*, IL-4, LTB4, LTC4, PGE2, cytosolic phospholipase A2, 5-lipoxygenase and COX-2, Akt, ERK, p38, JNK, PKC-*δ*, PLC*γ*1, Lyn, and Syk, but not much effect on Fyn	↓ MAPK, PKC-*δ*, PLC*γ*1, Lyn, Syk, Akt
Li et al., 2022 [11]	Mechanism of Schischk (Sk) on type I allergy	Sk; KF (+ve), POG (model group)	Anti-DNP IgE @ 0.4 μg/mL (12 h); Sk @ 0.5–2 mg/mL or POG @ 10–80 μg/mL or KF @ 30 μM (12 h); DNP-BSA @ 0.4 μg/mL (1 h)	IgE > Tmt > DNP-BSA	β-hex assay, RT-qPCR	SD ↓ β-hex, proteins in Lyn/Syk, PI3/AKT and MAPK	↓ Lyn/Syk, PI3/AKT and MAPK
Liu et al., 2021 [55]	Mechanism of sodium sulfite-induced pyroptosis and its effect on degranulation	Sodium sulfite; NAC or MCC950 (+ve)	Sodium sulfite @ 2–8 mM (30 min) or NAC @ 5 mM or MCC950 @ 10 μM (24 h) before treated with sodium sulfite @ 8 mM (30 min)	NAC orMCC950 > Sodium sulfite	β-hex assay, histamine assay, WB, MTT assay, ELISA	Sodium sulfite ↑ ROS, NLRP3, caspase-1, GSDMD-N, IL-1β, IL-18, cell membrane rupture, β-hex, histamine	↑ NLRP3, caspase-1
Liu et al., 2023 [9]	Viridicatol’s from *Penicillium griseofulvum* cell activation mechanism	Viridicatol; DNP-BSA (+ve)	Anti-DNP IgE @ 100 ng/mL (16 h); viridicatol @ 10 μg/mL (or @ 2.5–10 μg/mL for proteins JNK, ERK, p38, STAT6 study) (1 h); DNP-BSA @ 500 ng/mL (1 h) (or 15 min for protein measurement)	IgE > Tmt > DNP-BSA	RT-qPCR, RNA sequencing, WB	Viridicatol ↓ cell activation related genes *Tnfα, Ccl2, Jun, Fos, Il4, Ccl7, Il13,* and *Socs1* and proteins JNK, ERK, P38, and STAT6	↓ JNK, ERK, P38, and STAT6
Lv et al., 2022 [56]	Effect of ribose treated tropomyosin (from shrimp) (TM) on allergenicity	TM, glycated TM; Growth media (control)	Mouse sera @ 1:10 dilution (overnight); TM and 100 μL glycated TM @ 4000 mmol/l ribose (1 h)	Mouse Sera > Tmt	β-hex assay, ELISA	The glycated TM ↓ the histamine and β-hex	NA
Ma et al., 2022 [10]	Effect of POS on mast cell activation and degranulation	Oligosachharides; KF (+ve)	Anti-DNP IgE @ 0.5 μg/mL (overnight); galacturonic acid (GalA), Di-GalA, Tri-GalA or POS @ 75–300 μg/mL or KM (1 h); DNP-BSA @ 10 μg/mL or with PIPES @ 200 μL (30 min). For intracellular Ca^2+^ measurement, cells treated with inhibitors U73122, 2-APB, SKF96365 @ 19 μL (30 min); anti-DNP IgE; Tri-GalA @ 150 μg/mL; DNP-BSA	IgE > Tmt > DNP-BSA	β-hex assay, histamine release assay, ELISA, MTT assay	Tri-GalA and POS ↓ histamine, β-hex, IL-4, and Ca^2+^ influx	NA
Matsui et al., 2015 [57]	Anti-allergic effect of licarin A (a neolignan from plants)	Licarin; DNP-hSA (+ve)	Anti-DNP IgE @ 0.5 μg/mL (24 h); licarin A @ 0–20 μM (1 h) or only 20 μM (30 min) for Ca^2+^ measurement); DNP-hSA @ 0.2 μg/mL (30 min for histamine or (6 h) for PGD2 and COX-2 protein or (12 h) for TNF-*α*. For (p65, NF-*κ*B, p38 MAPK, JNK, PKC*α*/β II proteins, licarin A @ 20 μM; DNP-HSA (0–30 min). For mRNA of COX-2 and TNF-*α*, sensitized and treated cell were stimulated with DNP-hSA (1–5 h)	IgE > Tmt > DNP-hSA	Spectro fluorometry, MTT assay, ELISA and WB, PGD2 assay	Licarin A ↓ TNF-*α*, PGD2, COX-2 (mRNA level), PKC*α*/β II and p38 MAPK proteins	↓ PKC*α*/β II and p38 MAPK
Matsui et al., 2022 [58]	Anti-allergic activity of phlorotannins from brown seaweed	Phlorotannin; DNP-hSA (control)	Anti-DNP IgE @ 100 ng/mL (overnight); phlorotannins @ 0.2–300 μM (30 min); DNP-hSA @ 100 ng/mL (1 h) for β-hex or (8 h) for PGD2 and TNF-*α* or (1 h and 3 h) for mRNA of COX-2 and TNF-*α* or (10 h) for ROS	IgE > Tmt > DNP-hSA	β-hex assay, ELISA, MTT assay, PGD2 assay, WB	Phlorotannins ↓ β-hex, PGD2, TNF-*α*	NA
Mwakalukwa et al., 2019 [6]	Anti-allergic activity of olive mill waste (OMW) polyphenolic compounds	OMW extracts; DNP-BSA or A23187 (control)	Fractions of extracts such as new HDOA, luteolin, HOTy acetate @ 3.125–100 μg/mL or HOTy and 1-acetoxypin @ 5–250 μg/mL or DMSO @ 0.5 μL/well or quercetin (1 h); A23187 @ 5μM (1 h) or A23187 @10 μg/mL or DNP-BSA@ 100 ng/mL (180 sec) for Ca^2+^ measurement. For mRNAs of calcium channel proteins, TRPC1, STIM1, and Orai1 and ER membrane protein, IP3R, anti-DNP IgE sensitized cells were treated with compounds (16 h); DNP-BSA	Tmt> A23187;IgE > Tmt > DNP-BSA	RT-qPCR, β-hex assay, Calcium kit, MTT assay	Novel compound of OMW ↓ intracellular Ca^2+^ influx and calcium channel proteins	NA
Niu et al., 2020 [59]	Narirutin’s inhibition mechanism on degranulation	Narirutin; anti-DNP-IgE/DNP-BSA (control)	Anti-DNP IgE @ 0.5 μg/mL (overnight) or for Ca^2+^ (12 h); narirutin @ 0–200 μM (or 0–100 μM for histamine, Ca^2+^, IL-4 and TNF-*α* or 0–80 μM for mRNA of FcεRI *α*/β/*γ*) (2.5 h); DNP-BSA @ 0.5 μg/mL (1 h)	IgE > Tmt > DNP-BSA	CCK-8 assay, ELISA, WB, PCR, microscopy, β-hex assay, histamine assay	Narirutin ↓ Ca^2+^ influx via ↓ Syk, LAT, PLC*γ*1, and ↓ Ca^2+^ causes ↓ NF-κB. Narirutin also ↓ phosphorylated P38, ERK, JNK leading to ↓ of IL-4, TNF-*α*, histamine and β-hex	↓ MAPK family (p38, ERK, JNK), Syk, LAT, PLC*γ*1, and NF-κB
Shi et al., 2016 [23]	The anti-allergic activity of the Edulis Superba root extract	Extract; baicalein (+ve)	Anti-DNP IgE @ 0.5 μg/mL (24 h); extract @ 50 μM (30 min); DNP-BSA @ 1 μg/mL (1 h) and β-hex was measured	IgE > Tmt > DNP-BSA	β-hex assay, CCK-8 assay	The extract (mudanpioside E and quercetin) ↓ β-hex	NA
Vo et al., 2018 [60]	Anti-allergic property of brown algae extract (fucofuroeckol-A)	Fucofuroeckol-A (F-A); no UVB (-ve)	F-A @ 0–50 μM (24 h); UVB exposure (1 h) for ROS and histamine or (2 h) for cytokines or (10 min) for Ca^2+^	Tmt > UVB	ELISA, histamine assay, microscopy, MTT assay	F-A ↓ histamine, Ca^2+^, IL-1β, TNF-*α*, ROS	NA
Vo et al., 2020 [18]	Myricetin’s (from Aiton fruit) effect on mast cell activation	Myricetin; DNP-BSA (Control)	Myricetin @ 10–40 μM or only 40 μM for Ca^2+^ (24 h); anti-DNP-IgE @ 1μg/mL (10 min); DNP-BSA @ 1 μg/mL (1 h) or 10 min for Syk, PLC*γ*1, and NF-κB, or 24 h for IL-4, TNF-*α*. For DPPH and ABST assays, 100 μL of myricetin @ 2–16 μM were used	Tmt > IgE > DNP-BSA	MTT assay, WB, β-hex assay, ELISA, DPPH scavenging assay, ABTS scavenging assay	Myricetin ↓ β-hex and Ca^2+^, IL-4, TNF-*α*, Syk, PLC*γ*1, NF-κB, DPPH and ABTS+ radicals	↓ Syk, PLC*γ*1, NF-κB
Yan et al., 2024 [12]	Anti-allergic property of sea buckthorn flavonoid (SBF) or its purified form (PSBF)	SBF or PSBF; KM (+ve)	Anti-DNP-IgE @ 0.5 μg/mL (overnight) or 12 h for Ca^2+^; SBF or PSBF @ 25–100 μg/mL or five flavonoids of SBF/PSBF @ 10–40 μg/mL (1 h); DNP-BSA @ 10 μg/mL (30 min). For protein analysis, the five compounds, and SBF and PSBF were used @ 100 and 40 μg/mL, respectively	IgE > Tmt > DNP-BSA	β-hex assay, histamine release assay, WB, MTT assay, ELISA	PSBF ↓ degranulation, IL-4, extracellular Ca^2+^ influx, SBF ↓ p38, and JNK expression	↓ p38 and JNK expression
Zeng et al., 2023 [61]	Anti-allergic activity of curcumin and EGCG	Curcumin or EGCG; anti-DNP-IgE/DNP-BSA (+ve)	Anti-DNP-IgE @ 200 ng/mL (18 h); (curcumin @ 5–50 μM or 5–30μM for IL-4 and TNF-*α* or EGCG @ 100–650 μM or @ 100–500 μM for IL-4 and TNF-*α*)+ DNP-BSA @ 500 ng/mL (1 h or 3 h) or (3 h) for IL-4 and TNF-*α*	IgE > Tmt + DNP-BSA	β-hex assay, MTT assay, and ELISA	Both curcumin and EGCG ↓ β-hex, IL-4, and TNF-*α*	NA
Zhao et al., 2022 [62]	Mechanism of *Paeonia lactiflora* Pall (PLP) on anti-allergic effect	Paeoniflorin; KF (+ve)	Anti-DNP-IgE @ 0.2 μg/mL (12 h); Paeoniflorin @ 0.5–5 μg/mL or KF @ 25 μg/mL (1 h); DNP-BSA @ 0.4 μg/mL (30 min)	IgE > Tmt > DNP-BSA	WB, RT-qPCR	Paeoniflorin ↓ Lyn, Syk, Fyn, PLC*γ*, PI3K, Akt, p38, ERK, JNK, and p65 genes	↓ Lyn, Syk, Fyn, PLC*γ*, PI3K, Akt, p38, ERK, JNK, and p65

↓: decrease/inhibit expression; ↑: increase/promote expression; ABTS 2,2′-Azino-bis(3-ethylbenzthiazoline-6-sulfonic acid); Akt: protein kinase B; β-hex: β-hexosaminidase; CCK-8: cell counting kit-8; COX-2: cyclooxygenase-2; CSV: crystal violet staining assay; DMSO: dimethyl sulfoxide; DNP-BSA: 2,4-dinitrophenyl-bovine serum albumin; DNP-HSA: 2,4-dinitrophenyl- horse serum albumin; DNP-hSA: 2,4-dinitrophenyl- human serum albumin; DPPH: 2,2-diphenyl-1-picrylhydrazyl Radical; DTBHQ: 2,5-*ditert*-butylhydroquinone; EGCG: epigallocatechin gallate; ELISA: enzyme-linked immunosorbent assay; ER: endoplasmic reticulum; ERK: extracellular signal-regulated kinase; FBS: fetal bovine serum; GSDMD-N: gasdermin D N-terminal; HPLC: high-pressure liquid chromatography; IgE: anti-DNP-immunoglobulin E; IL: interleukin; IP3R: inositol-1, 4, 5-triphosphate receptor; KF: ketotifen fumarate; LAT: linker for activated T cell; Lyn: src-family kinase; MAPK: mitogen-activated proteins kinase; MCC950: NLRP3 inhibitor; MEM: minimal essential medium; MTT: 3-[4,5-dimethylthiazol-2-yl]-2,5 diphenyl tetrazolium bromide; NA: not applied/available; NAC: N-actyl-L-carnosine (ROS scavenger); NF-κB: nuclear factor kappa B; NLRP3: NOD-like receptor protein 3; ORAC: oxygen radical absorbance capacity; Orai1: calcium release-activated calcium channel protein 1; P38: P38 MAPK; PGD2: prostaglandin D2; PI3K: phosphoinositide 3-kinase; PKC: protein kinase C; PLCγ: phospholipase C; PMA: phorbol 12-myristate 13-acetate; PMF: polymethoxyflavones; PN: peanut; POG: prim-O-glucosylcimifugin; POS: pectic oligosaccharides; PSP: peanut skin polyphenol; RBL-2H3: rat basophilic leukemia cell line; ROS: reactive oxygen species; RT-qPCR: real-time quantitative polymerase chain reaction; SAPK: stress-activated protein kinase; JNK: c-Jun terminal kinase; SARS: superoxide anion radical scavenging; STAT6: signal transducers and activators of transcription; STIM1: stromal interaction molecule 1; Syk: spleen tyrosine kinase; Tmt: treatment; TNF-α: tumor necrosis factor alpha; TPC: total phenolic content; TRPC1: transient receptor potential channel 1; UVB: ultraviolet B; WB: Western blot.

**Table 2:** Anti-allergic and anti-inflammatory properties and signaling pathways of various compounds using RBL-2H3 cell and other cell lines.

Ref.	Objectives	Methods: Cell Line	Methods: Treatment; Control	Methods: Dose (Duration)	Method: Sensitization, Treatment, Stimulation Sequence	Methods: Assays	Main Findings	Findings: Signaling Pathways in RBL-2H3 Cells
Badger-Emeka et al., 2020 [1]	Anti-allergic effect of cinnamaldehyde (CA) from cinnamon bark	RBL-2H3, *Klebsiella pneumoniae* (bacteria)	CA; DMSO (control),DNP-IgE (control)	For RBL-2H3: CA or DMSO @ 5–50 µM (10 min); DNP-IgE (24 h); DNP-BSA (24 h). For bacteria, the 25 µL of 5–50 µM CA dissolved in 0.1% DMSO was applied to bacterial culture and incubated at 37 °C (12 h), and inhibition zones for HDC activity were measured.	Tmt > IgE > DNP-BSA	MTT assay, ELISA, WB, RT-qPCR, histamine assay, and β-hex assay	CA ↓ MAPK p38/ERK pathway. CA ↓ all the measured parameters compared to the DNP-IgE treated RBL-2H3 cells. CA ↓ the HDC activities both in RBL-2H3 and bacterial cells	↓ MAPK p38/ERK
Dera et al., 2020 [15]	Effect of thymoquinone from black caraway on allergy	RBL-2H3, RAW264.6, human neutrophil basophil	Thymoquinone (Tq); anti-DNP-IgE + DNP-BSA (control)	For RBL-2H3: Anti-DNP IgE @ 1 μg/mL (overnight); Tq @ 0–50 μM (30 min) or 1 h for degranulation and cytokines; DNP-BSA @ 0.025 μg/mL (4 h).	IgE > Tmt > DNP-BSA	BAT assay, MTT assay, neutrophil migration assay, neutrophil elastase assay, ELISA, WB	Tq dose-dependently ↓ of TNF-α and IL-4. Tq ↓ Akt, NF-κB phosphorylation, and ↑ nuclear Nrf2 and HO-1 proteins in activated RBL-2H3 cells	↓ NF-κB and Akt
Hanieh et al., 2017 [63]	Pinocembrin’s effect on IgE-mediated response	RBL-2H3, *Klebsiella pneumoniae* (bacteria)	Pinocembrin; DMSO (+ve)	For RBL-2H3: DMSO or Pinocembrin @ 10–50 μmole/l (10 min); anti-DNP-IgE @ 1 μg/mL (4 h) for cell viability test or 24 h for β-hex, NO, WB and ELISA; DNP-BSA @ 100 μg/mL (24 h). For bacteria and microbial integrity tests, the sensitized and stimulated cells were stained with rhodamine123 @ 1 μg/mL. The bacteria were also used for a preliminary study of HDC activity.	Tmt > IgE > DNP-BSA	β-hex assay, WB, RT-qPCR, HDC activity and inhibitory activity assays, MTT assay, MMIT, NO assay	Pinocembrin ↓ HDC activity, histamine, damage of mitochondrial membrane, β-hex, TNF-α, IL-6, iNOS, PGE-2, and COX-2. Pinocembrin ↑ p38 MAPK through IkB pathway	↑ p38 MAPK through IkB pathway
Hagenlocher et al., 2015 [19]	Cinnamaldehyde’s (CA) or Cinnamon extract (CE) effect on mast cell activation	RBL-2H3, human intestine mast cell (hiMC)	CA, CE; 0.1% DMSO (control for CA), 70% ethanol (control for CE)	For RBL-2H3 and hiMC cells: CA @ 5– 500 μM or CE @ 1 and 10 μL/mL or DMSO or 70% ethanol (18 h). (a) for IgE-dependent activation: DNP-IgE for RBL-2H3 or myeloma IgE for hiMC @ 0.1 μg/mL; DNP-BSA (RBL-2H3) @ 0.1 μg/mL or polyclonal anti-human IgE (hiMC) (5 min) or (10 min) for ERK and PLC*γ*1 or (90 min) for cytokine and mediator release or (6 h) for CXCL8. (b) For IgE-independent activation, cells were stimulated with ionomycin/PMA) @ 1 μM.	Tmt > IgE > DNP-BSA;Tmt > myeloma IgE > Anti-human IgE; Tmt > IgE > Ionomycin or PMA	Cell death detection kit, ELISA, β-hex assay, RT-PCR, WB	In RBL-2H3, CA ↓ the β-hex in dose-dependent manner to 10%. In hiMC, CA ↓ β-hex, LTC4, CXCL8, CCL2–4, ERK and PLC*γ*1	NA
Kong et al., 2020 [22]	Curcumin’s effect on allergic inflammation	RBL-2H3 and huma pre-basophils (KU812)	Curcumin; no curcumin, no IgE, or no A23187(controls)	For RBL-2H3: anti-DNP IgE @ 500 ng/mL (24 h) or @ 1 μg/mL (overnight) for histamine or ROS, respectively; curcumin @ 1–30 μM; DNP-BSA @ 500 ng/mL (1 h) or (13 min) for ROS. For A23187 dependent activation: curcumin: A23187 @ 1μM or @ 2 μM for histamine or ROS, respectively (30 min) or ROS (13 min). For RT-PCR, KU812; for PKC, RBL-2H3; and for FcεRI protein, both cell types were used.	IgE > Tmt > DNP-BSA or A23187	β-hex assay, WB, MTT assay, histamine release assay, ROS production assay, RT-PCR	Curcumin ↓ β-hex, histamine, ROS, FcεRI, IL-4, IL-13 production, and PKC-*δ* translocation in IgE or A23187 induced cells	↓ FcεRI and PKC-*δ*
Korinek et al., 2016 [45]	Mechanism of the anti-allergic and anti-inflammatory effect of *Typhonium blumei* and *T. roxburghii*	RBL-2H3 and human neutrophil	Extracts (TBLE or TBE); dexamethasone (+ve)	For RBL-2H3: extracts (TBLE or TBE) @ 10–100 μg/mL or TBLE @ 1–100 μg/mL for mRNA study (20 h); (a) for IgE independent activation: A23187 @1 μM (1 h) or 2 μM (10 min) for proteins measurement) or (b) for IgE-dependent activation: anti-DNP IgE @ 5 μg/mL (2 h); DNP-BSA @ 100 ng/mL (1 h) or (10 min) for mRNA and protein study. Protein of ERK, JNK, p-38, Akt, PI3K and PLC*γ*2, and mRNA of IL-4 and MCP-1 were measured.	Tmt > IgE > A23187 or DNP-BSA	β-hex assay, WB, MTT assay, histamine release assay, flow cytometry, fluorescence microscopy, RT-qPCR, SAG assay	*T. blumei*’s non-polar fraction ↓ antigen-induced β-hex, histamine, and calcium influx (induced by antigen and A23187). No effect on FcεRI, IL-4, and MCP (mRNA) or MAPK, but ↓ PI3K/PLC*γ*2	↓ PI3K/PLC*γ*2
Lim et al., 2023 [27]	Therapeutic potential of *Rosa davurica* leaf extract (RLE) against allergy	RBL-2H3 and RAW 264.7	RLE; KF and tacrolimus (+ve control for RBL-2H3), dexamethasone (+ve control for RAW 264.7)	For RBL-2H3: anti-DNP IgE @ 50 ng/mL (24 h); RLE @ 10–100 μg/mL or KM @ 50 μM or tacrolimus @ 50 ng/mL (1 h); DNP-BSA @100 ng/mL (4 h). For RAW 264.7: RLE @ 10–100 μg/mL (1 h); LPS @ 1 μg/mL or dexamethasone @ 10 μM. In RAW 264.7, mRNA of inducible nitrogen oxygen synthase (iNOS), IL-1β, Il-6, TNF-*α*, COX-2, and NO were measured.	IgE > Tmt > DNP-BSA; Tmt > LPS	β-hex assay, histamine assay, WB, MTT assay, ELISA, calcium assay, RT-qPCR	In Raw 264.7, RLE ↓ NO, COX-2, iNOS, IL-1β, Il-6, TNF-*α*. In RBL-2H3, RLE ↓ β-hex, histamine, HDC, Ca^2+^ influx, Ca^2+^ pathways, and MAPK	↓ MAPK (p38, JNK, ERK)
Lorenz et al., 2016 [5]	Effect of oak bark decoction (OBD) and tannin in degranulation	RBL-2H3 and human mast cell (HMC-1)	OBD tannin fractions; DMEM (-ve control), azelastine (+ve control) for RBL-2H3; DMEM and dexamethasone (+ve controls) for HMC-1	For RBL-2H3: anti-DNP IgE @ 0.5 μg/mL (12–18 h); OBD tannin fractions A @ 0.017–0.17 mg/mL or fractions (B–D) @ 6–100 μg/mL (10 min); DNP-hSA @ 20 ng/mL DNP-HSA (30 min). For HMC-1: OBD fractions A–D at the same rate as for RBL-2H3 cells (30 min) or dexamethasone; PMA @ 40 nM and A23187 @ 1 μM (2.5 h) and IL-8, IL-6, and TNF-*α* were measured.	IgE > Tmt > DNP-hSA; Tmt > PMA + A23187	WST-1 assay, β-hex assay, ELISA	The fractions ↓ β-hex (in RBL-2H3), IL-8, IL-6, and TNF-*α* in a dose-dependent manner	NA
Min et al., 2021 [26]	Anti-inflammatory, ani-allergic properties of saponarin from green barley leaves	RBL-2H3, RAW264.7, Human immortalized keratinocyte (HaCaT) cells	Saponarin; cyclosporine A (control for RBL-2H3), quercetin (control for RAW264.7)	For RAW264.7: LPS @ 1 μg/mL; saponarin @ 20–80 μM (or only 80 μM for ERK, JNK, p38, TNF-*α*, IL 6, IL-1β, iNOS, COX-2) or quercetin @ 15μM+ LPS @ 1 μg/mL (24 h) and NO was measured. For RBL-2H3: anti-DNP IgE @ 0.5 μg/mL (24 h); saponarin @ 5–40 μM for β-hex or only 40 μM for rest of all measurements or cyclosporine A @ 1 μg/mL (20 min); DNP-BSA @ 100 ng/mL (1 h). For HaCaT: 100 μM saponarin (24 h) or (1 h for ERK, JNK, p38) or (18 h for IL-33, IL-25, MDC, TARC, TSLP); 50 ng/mL of TNF-*α* and IFN-*γ* (24 h) (or 15 min for ERK, JNK, p38 or 6 h for IL-33, IL-25, MDC, TARC, TSLP) and STAT1 was measured.	LPS > Tmt;IgE > Tmt > DNP-BSA; Tmt > TNF-*α* and IFN-*γ*	β-hex assay, ELISA, MTT assay, RT-qPCR, WB	Saponarin (80 μM) ↓ TNF-*α*, IL-1β, iNOS, COX-2, ERK and p38 MAPK in RAW264.7 cells. Saponarin (40 μM) ↓ β-hex, Syk, PLC*γ*1, ERK, JNK, p38, TNF-*α*, IL-4, 5, 6, 13, COX-2, and FcεRI *α*/*γ* in RBL-2H3. Moreover, Saponarin (100 μM) ↓ IL-33, ERK, p38, STAT1, in HaCaT cells	↓ FcεRI *α*/*γ*, PLC*γ*1 and MAPK family (ERK, JNK, p38)
Park et al., 2020 [16]	Anti-allergic effect of barley sprout extract (apigenin) on RBL-2H3, anti-inflammatory effect on RAW264.7 and AD potential on HaCaT cells	RBL-2H3, RAW264.7, Human epidermal keratinocyte (HaCaT) cells	Apigenin; cyclosporine A (control for RBL-2H3), quercetin (control for RAW264.7).	For RAW264.7: LPS @ 1 μg/mL and apigenin @ 20–100 μM (or only 100 μM or 15μM quercetin for cytokines and MAPK signaling proteins) (24 h) and NO was measured. For RBL-2H3: anti-DNP-IgE @ 0.5 μg/mL (24 h); apigenin @ 5–30 μM (or only 30 μM or cyclosporine A @ 1 μg/mL for mRNA of cytokines, FcεRI *α*, MAPK proteins) (20 min); DNP-BSA @ 100 ng/mL (1 h) and β-hex was measured. For HaCaT: apigenin @ 20 μM (24 h) and genes related to skin physical and chemical barrier function were measured.	Tmt + LPS; IgE > Tmt > DNP-BSA; Tmt	MTT assay, WB, ELISA, β-hex assay, RT-qPCR	In RAW264.7 cells, 100 μM apigenin ↓ NO, IL-1β, IL6, COX-2, iNOS, ERK, JNK. In RBL-2H3 cells, 30 μM apigenin ↓ Lyn, Syk, PLC*γ*1, ERK, JNK, FcεRI *α*, TNF-*α*, IL-4, -5, -6, and COX-2. In HaCaT cells, 20μM apigenin ↑ gene/protein of compounds related to chemical and physical barrier of skin	↓ Lyn, Syk, PLC*γ*1, ERK, JNK, FcεRI *α*
Yoo et al., 2017 [17]	Coumarin derivative’s effect on mast cell degranulation	RBL-2H3, RAW264.7, MOLT-4	Coumarin derivative 1 (C1); Loratadine (+ve control for RBL-2H3), LPS (+ve control for RAW264.7)	For RBL-2H3: C1 or loratadine (not for protein study) @ 0–25 μM (1 h); PMA @ 50 nM+ A23187 @ 1 μM (30 min) and β-hex, histamine, p38, ERK, JNK, MKK3, MEK1/2, and MKK4 were analyzed. For RAW264.7: C1 @ 0–25 μM (24 h) for NO production or (1 h for NO inhibition); LPS @ 1μg/mL (24 h). For MOLT-4: C1 0–25 μM (6 h) and mRNA of IL-4 and IFN-*γ* measured.	Tmt > PMA + A23187;Tmt > LPS; Tmt	β-hex assay, histamine release assay, WB, RT-qPCR, nitrite assay, MTT assay	C1 ↓ β-hex, histamine, and ERK with maximum effect at 25 μM	↓ ERK
Yoshioka et al., 2020 [13]	Anti-allergic and anti-inflammatory effects of ESG	RBL-2H3, human epithelial cell lineage (Caco-2), BMMC	ESG; DMEM media (-ve control)	For RBL-2H3 or BMMC cells: Anti-DNP-IgE @ 1 μg/mL (16 h); ESG @ 100–1000 μg/mL (in co-culture system treated to Caco-2) (24 h); DNP-BSA @ 10 ng/mL (30 min) (in co-culture system, only RBL-2H3 and BMMC were sensitized and challenged).	IgE > Tmt > DNP-BSA	β-hex assay, WB, ELISA	ESG ↓ β-hex, TNF-*α*, IL-6, Lyn, Syk, PLC*γ*1/2, MAPK, and Akt in RBL-2H3 of co-culture system	↓ Lyn, Syk, PLC*γ*1/2, MAPK and Akt

↓: decrease/Inhibit expression; ↑: increase/promotes expression; A23187: calcium ionophore; AD: atopic dermatitis; Akt: protein kinase B; BAT: basophil activation test; BMMC: bone marrow mononuclear cells; Caco-2: colon tissue epithelial cells; CCL: CC chemokine ligand; COX-2: cyclooxygenase-2; CXCL8: c-x-c motif chemokine ligand 8; DMEM: Dulbecco’s modified eagle medium; DMSO: dimethyl sulfoxide; DNP-BSA: 2,4, dinitrophenyl bovine serum albumin; DNP-hSA: 2,4-dinitrophenyl- human serum albumin; ELISA: enzyme-linked immunosorbent assay; ERK: extracellular signal-regulated kinase; ESG: enzymatically synthesized glycogen; HDC: histidine decarboxylase; HO-1: heme-oxygenase 1; IgE: Anti-DNP-Immunoglobulin E; IkB: inhibitor of kappa B; MAPK: mitogen activated proteins kinase; MDC: macrophage-derived chemokine; MMIT: mitochondrial membrane integrity test; MOLT-4: T lymphoblast cell line; MTT: 3-[4,5-dimethylthiazol-2-yl]-2,5 diphenyl tetrazolium bromide; NO: nitric oxide; Nrf2: nuclear factor erythroid 2- related factor 2; P38: P38 MAPK; PKC: protein kinase C; PLCγ: phospholipase Cγ; RAW264.6: mouse macrophage cell line; RBL-2H3: rat basophilic leukemia cell line; ROS: reactive oxygen species; RT-qPCR: real time quantitative polymerase chain reaction; SAG: superoxide anion generation; STAT: signal transducers and activators of transcription; TARC: thymus and activation-regulated chemokine; Tmt: treatment; TNF-*α*: tumor necrosis factor alpha; TSLP: thymic stromal lymphopoietin; WB: Western blot; WST: water soluble tetrazolium.

**Table 3:** Quality assessment of reviewed studies according to modified CONSORT checklist [49,50].

Ref.	Abstract	Introduction			Methods			Results	Discussion	Other Info.
	1	2a	2b		3	4	10	11	12	13
		Background and Rationale	Objectives	Hypothesis	Intervention	Outcomes	Statistical Methods	Outcomes and Estimations	Limitations	Funding
Badger-Emeka et al., 2020 [1]	Yes	Yes	Yes	No	Yes	Yes	Yes	Yes	No	Yes
Bansode et al., 2018 [51]	Yes	Yes	Yes	Yes	Yes	Yes	Yes	Yes	No	Yes
Barbosa et al., 2018 [2]	Yes	Yes	Yes	No	Yes	Yes	Yes	Yes	No	Yes
Chang et al., 2015 [3]	Yes	Yes	Yes	No	Yes	No	Yes	Yes	No	Yes
Dera et al., 2020 [15]	Yes	Yes	Yes	No	Yes	Yes	No	Yes	No	Yes
Dippenaar et al., 2022 [52]	Yes	Yes	Yes	No	Yes	Yes	Yes	Yes	Yes	Yes
Gaihre et al., 2022 [53]	Yes	Yes	Yes	No	Yes	Yes	Yes	Yes	No	Yes
Hamauzu et al., 2021 [54]	Yes	Yes	Yes	No	Yes	Yes	No	Yes	No	Yes
Hanieh et al., 2017 [63]	Yes	Yes	Yes	Yes	Yes	Yes	Yes	Yes	No	Yes
Hagenlocher et al., 2015 [19]	Yes	Yes	Yes	Yes	Yes	Yes	Yes	Yes	No	No
Jiao et al., 2017 [20]	Yes	Yes	Yes	No	Yes	Yes	Yes	Yes	No	Yes
Kawai et al., 2018 [21]	Yes	Yes	Yes	No	Yes	Yes	Yes	Yes	Yes	Yes
Kobayashi et al., 2015 [4]	Yes	Yes	Yes	No	No	No	Yes	Yes	No	Yes
Kong et al., 2020 [22]	Yes	Yes	Yes	No	No	No	Yes	No	No	Yes
Korinek et al., 2016 [45]	Yes	Yes	Yes	No	No	Yes	Yes	Yes	No	Yes
Lee et al., 2017 [8]	Yes	Yes	Yes	No	No	No	Yes	Yes	No	Yes
Lee et al., 2020 [25]	Yes	Yes	Yes	No	Yes	Yes	Yes	Yes	Yes	Yes
Li et al., 2022 [11]	Yes	Yes	Yes	No	Yes	Yes	Yes	Yes	No	Yes
Lim et al., 2023 [27]	Yes	Yes	Yes	No	Yes	Yes	Yes	Yes	No	Yes
Liu et al., 2021 [55]	Yes	Yes	Yes	Yes	Yes	Yes	Yes	Yes	No	Yes
Liu et al., 2023 [9]	Yes	Yes	Yes	No	Yes	Yes	Yes	Yes	No	Yes
Lorenz et al., 2016 [5]	Yes	Yes	Yes	No	Yes	Yes	Yes	Yes	No	Yes
Lv et al., 2022 [56]	Yes	Yes	Yes	Yes	Yes	Yes	Yes	Yes	No	Yes
Ma et al., 2022 [10]	Yes	Yes	Yes	No	Yes	Yes	Yes	Yes	No	Yes
Matsui et al., 2015 [57]	Yes	Yes	Yes	Yes	Yes	Yes	Yes	Yes	Yes	Yes
Matsui et al., 2022 [58]	Yes	Yes	Yes	No	Yes	Yes	Yes	Yes	No	Yes
Min et al., 2021 [26]	Yes	Yes	Yes	No	No	Yes	Yes	Yes	No	Yes
Mwakalukwa et al., 2019 [6]	Yes	Yes	Yes	Yes	No	Yes	Yes	Yes	Yes	Yes
Niu et al., 2020 [59]	Yes	Yes	Yes	No	Yes	Yes	Yes	Yes	No	Yes
Park et al., 2020 [16]	Yes	Yes	Yes	No	Yes	Yes	Yes	Yes	No	Yes
Shi et al., 2016 [23]	Yes	Yes	Yes	No	Yes	Yes	No	No	No	Yes
Vo et al., 2018 [60]	Yes	Yes	Yes	No	Yes	Yes	Yes	Yes	No	Yes
Vo et al., 2020 [18]	Yes	Yes	Yes	No	Yes	Yes	Yes	Yes	No	Yes
Yoo et al., 2017 [17]	Yes	Yes	Yes	No	Yes	Yes	Yes	Yes	No	Yes
Yan et al., 2024 [12]	Yes	Yes	Yes	No	Yes	Yes	Yes	Yes	No	Yes
Yoshioka et al., 2020 [13]	Yes	Yes	Yes	No	Yes	Yes	Yes	Yes	No	Yes
Zeng et al., 2023 [61]	Yes	Yes	Yes	No	Yes	Yes	Yes	Yes	No	Yes
Zhao et al., 2022 [62]	Yes	Yes	Yes	Yes	Yes	Yes	Yes	Yes	No	Yes

## Data Availability

Not applicable.
